# Automatic segmentation tool for 3D digital rocks by deep learning

**DOI:** 10.1038/s41598-021-98697-z

**Published:** 2021-09-27

**Authors:** Johan Phan, Leonardo C. Ruspini, Frank Lindseth

**Affiliations:** 1 Department of Computer Science, NTNU, Trondheim, Norway; 2 Petricore Norway, Trondheim, Norway; 3 Codego AS, Trondheim, Norway

**Keywords:** Computer science, Hydrology, Carbon capture and storage

## Abstract

Obtaining an accurate segmentation of images obtained by computed microtomography (micro-CT) techniques is a non-trivial process due to the wide range of noise types and artifacts present in these images. Current methodologies are often time-consuming, sensitive to noise and artifacts, and require skilled people to give accurate results. Motivated by the rapid advancement of deep learning-based segmentation techniques in recent years, we have developed a tool that aims to fully automate the segmentation process in one step, without the need for any extra image processing steps such as noise filtering or artifact removal. To get a general model, we train our network using a dataset made of high-quality three-dimensional micro-CT images from different scanners, rock types, and resolutions. In addition, we use a domain-specific augmented training pipeline with various types of noise, synthetic artifacts, and image transformation/distortion. For validation, we use a synthetic dataset to measure accuracy and analyze noise/artifact sensitivity. The results show a robust and accurate segmentation performance for the most common types of noises present in real micro-CT images. We also compared the segmentation of our method and five expert users, using commercial and open software packages on real rock images. We found that most of the current tools fail to reduce the impact of local and global noises and artifacts. We quantified the variation on human-assisted segmentation results in terms of physical properties and observed a large variation. In comparison, the new method is more robust to local noises and artifacts, outperforming the human segmentation and giving consistent results. Finally, we compared the porosity of our model segmented images with experimental porosity measured in the laboratory for ten different untrained samples, finding very encouraging results.

## Introduction

Capturing microscopic properties of porous rocks and their interaction with fluids play an instrumental role in many important domains such as carbon storage, oil and gas recovery, and underground water management. The use of 2D and 3D imaging techniques to capture these properties, known as Digital Rock Analysis (DRA), is becoming a common practice in the above-mentioned industrial applications^1–3^. However, in order to do any computation, the images need to be segmented into their constituent phases. Image segmentation is the process of labeling voxels into classes, which can be later used for the characterization of physical properties. Several segmentation techniques were developed in the past^4^. Due to the nature of the problem these tools include processes such as thresholding and clustering. However, these methods require a large degree of manual interaction and quality control. Some of the conventional methods are global multi-Otsu thresholding^5^, Marker-controlled Watershed^6^, and converging active contours. In addition, these methods require the use of different types of filters to deal with noise and artifacts. The recent advances in deep-learning technologies based on neural networks have led to the emergence of high-performance automatic segmentation techniques, with the highest accuracy rates on popular benchmarks^7^. For 3D images, deep learning-based segmentation techniques using Convolutional Neural Networks (CNNs)^8^ have been successfully used in many fields such as autonomous driving^9^, point cloud analysis^10^, medical image analysis^11,12^.

Recently, several forms of multivariant classifiers using machine learning have been used to segment 2D micro-CT rock images^13,14^. These last methods require the user to define/paint some areas with the desired labels to train with and then perform a segmentation of the whole image. The high degree of abstraction of deep learning methods has proven to be very effective compared to other segmentation techniques. In DRA, deep learning based segmentation has been successfully used on 2D binary segmentation^15^ and multi-mineral segmentation^16,17^. These last two works have explored the use of different CNNs architectures, such as SegNet^18^, ResNet, UNet, UResNet-3D, etc. These studies concluded that the more complex architecture U-ResNet-3D performed better for the majority of the cases in terms of accuracy and topological similarity properties. As reported in this last work, the authors used a single dataset (1100 $\times$ 1100 $\times$ 2200 voxels) for both training and testing. In this respect, due to the small difference in terms of image properties and gray-scale values within a single sample, it is expected that the more complex the network the higher probability of overfitting the problem. Additionally, as we have experienced, training and testing on the same image certainly lead to poor performance on untrained images and different types of rock.

Due to resolution limitations of current imaging technology (field of view vs. spatial resolution), a typical rock image contains a non-neglectful amount of sub-resolution porosity, i.e. the pores of these regions are below the image resolution. Therefore, a three-phase segmentation is necessary to account for the effect of these areas on flow properties^19–21^. In this work, unresolved porosity regions are also referred to as micro-phase.

The main goal of this work is to study the possibility of using a CNN model to perform automatic three-phase segmentation of real 3D Xray micro-CT images. This means:

1. No need for human intervention in the segmentation process.
2. Perform well with different rock types.
3. No filtering. Handling typical noises and artifacts, such as Gaussian noise, beam-hardening, ring artifacts.
4. Perform well with untrained data.
5. Preserve connectivity and continuity of the different regions (3D information).

## Materials and methods

In this work we have developed a new segmentation tool, using deep-learning, which specializes in three-phase segmentation. This tool and other AI tools for Digital Rocks are available under the SmartRocks project (smartrocks.com).

### Model architecture

**Figure 1:**
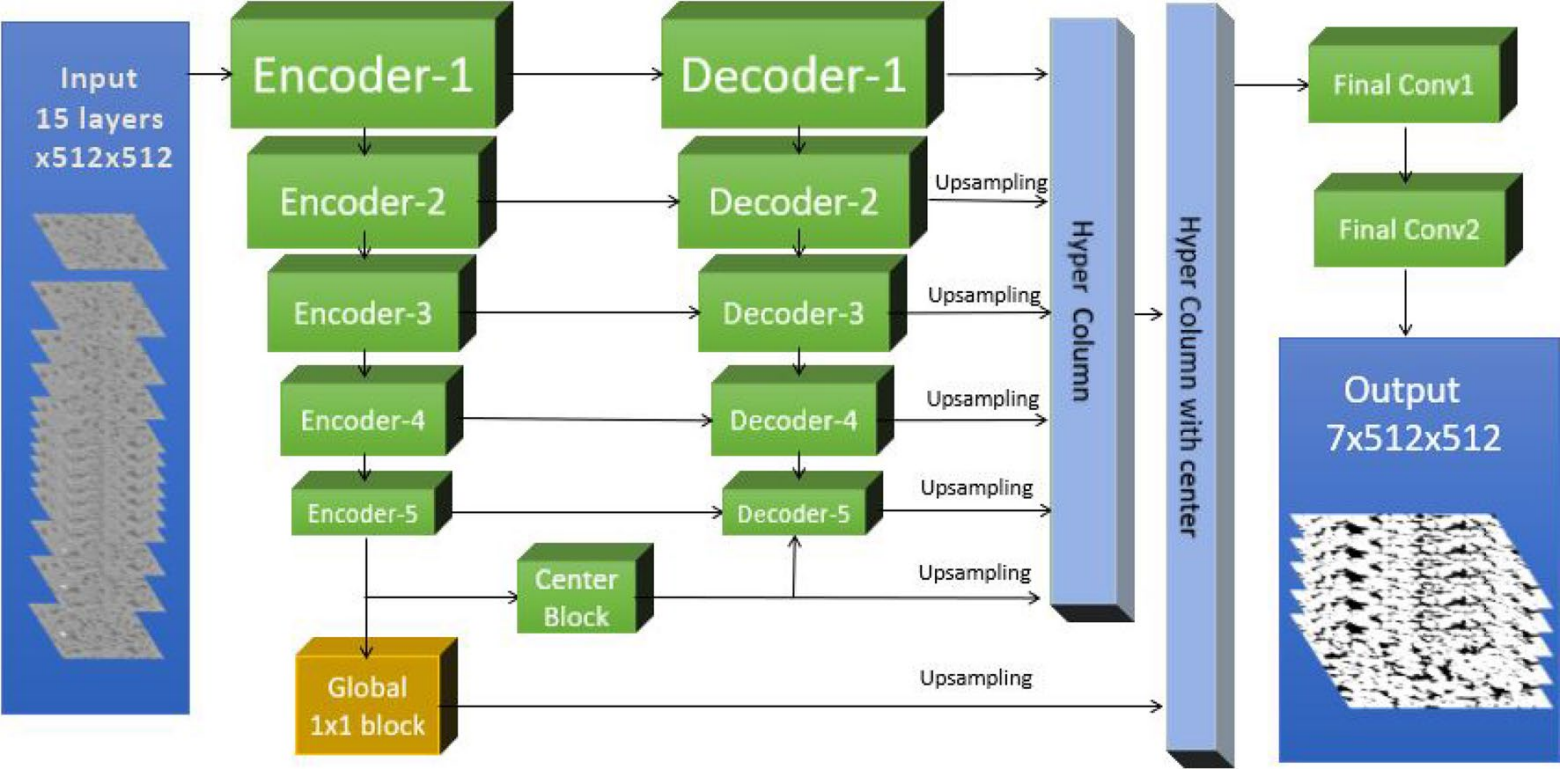
Architecture of the convolutional neural network model developed in this work. The model is based on an encoder-decoder architecture which takes a 3D image as input and split it in 2D slices (channels). In each iteration, the model takes 15 channels from 7 consecutive center layers and 8 skip layers as input. The model produces a 3D block of 7 consecutive channels as output.

The Neural Network architecture used in this work is inspired by the architecture used by many top teams in the TGS Salt Identification Challenge on Kaggle^22,23^. It is based on an improved version of the popular U-net architecture^12^ with SE-ResNeXt-50 encoder^24^ initialized with pre-trained parameters. A general view of all the components in this architecture is presented in Fig. 1. We used 2D convolutional blocks, that takes a stack of consecutive slices where each slice is treated as an input channel. In this way, using a 2D convolution allows us to decrease the computational cost and redirect the resources to train a more complex and deeper architecture. Moreover, we maximize the field of view on the X–Y plane (i.e. rotation plane), which is beneficial to deal with global noises and artifacts (e.g. ring artifacts, beam hardening) due to the nature of image acquisition. It also means that we can use pre-trained blocks trained with large datasets, such as the ImageNet dataset^25^. The common challenge of using 2D convolutional based network to segment 3D images is to preserve the depth information/connectivity of the orthogonal direction (Z direction in our case). To segment a 3D image, our model iterates over every slice of the image and takes a stack of slices containing the center slice and 7 slices in each direction, where 3 of them are consecutive and 4 are skip slices. Finally, our model produces a probability map for each phase, which is build as an average of 2D probability maps for each layer. The final segmentation is generated by selecting the phase with the highest probability for each voxel. To improve the model’s ability to interpret spatial information (cross-channel), we used a scSE block (Concurrent Spatial and Channel Squeeze and Excitation)^26^ as our decoder. In order to improve the output connectivity, a deep supervision block i.e., convolution + up-sample layers are added at the end of each decoder block to implement the hyper-column technique^27^. Another important part of our architecture is a global, center block made of a 1 $\times$ 1 convolutional block, which has a similar function to a dense block with fully connected layers. The goal of this intermediate global block is to give the network the capacity of a global classifier, to handle information such as rock type and non-local artifacts.

### Training dataset

Our training dataset consists of 10 3D Xray micro-CT images of different rock types. The properties of the images are listed in Table 1. These images were acquired using different procedures: scanners (e.g. Xradia, Heliscan), filters, exposure times, cleaning/cropping, reconstruction algorithms, etc. The main idea of using a diverse training dataset is to give our model the ability to generalize as much as possible. The ground truth segmentation for each of these images was obtained using different open and commercial software packages for filtering and segmenting (Avizo, Pergeos, ImageJ, Mango). In this way, we compensate for any systematic error in the acquisition, filtering, and segmentation procedures for our training data. All the selected images have low noise level and high-quality, which makes the segmentation step relatively easy. We have experienced that using segmentations from noisy images deteriorates the network’s ability to generalize and produces good results on untrained datasets. For the Reservoir carbonates and Savonnieres samples, the utilized segmented images were obtained from dry/wet based porosity maps^28,29^, which allow us to have a proper estimation of open and micro-phase porosities.

In addition, we have used a high degree of data augmentation to improve the robustness and generalization capabilities of the model. Besides the typical data augmentation techniques like rotating, random cropping, adding Gaussian noise and/or *Salt and Pepper* noise, we have also used more domain-specific noise in our training pipelines to match the real problems such as ring artifacts, stripe artifacts, intensity variations, and local blurring.

**Table 1:** Our training dataset consists of 10 high-resolution and low noise 3D Xray micro-CT images from different rock types.

Rock type	Voxel size ($\mathbf {X\times Y\times Z)}$			Resolution (µm)	Pore (%)	Micro (%)
	X	Y	Z			
Res. sandstone	792	764	1180	8.56	25.29	3.56
Bentheimer sandstone	1024	1024	1024	1.94	22.98	3.93
Res. sandstone	2560	2560	2840	3.46	16.6	6.98
Res. sandstone	2300	2300	2400	3.46	16.65	5.16
Glass bead^30^	550	550	572	8.41	32.99	0
Res. carbonate	1500	1500	3000	17.09	22.32	67.96
Res. carbonate	1500	1500	3000	17.09	11.28	73.52
Estaillades carbonate^31^	1200	1200	1700	3.10	6.43	39.24
Massangis carbonate^31^	501	408	600	4.54	4.53	13.82
Savonniers carbonate^20^	610	610	610	5.90	10.60	74.29

*Pore* and *Micro* are the volumetric fractions of resolved and unresolved porosity regions, respectively.

### Validation workflow

**Figure 2:**
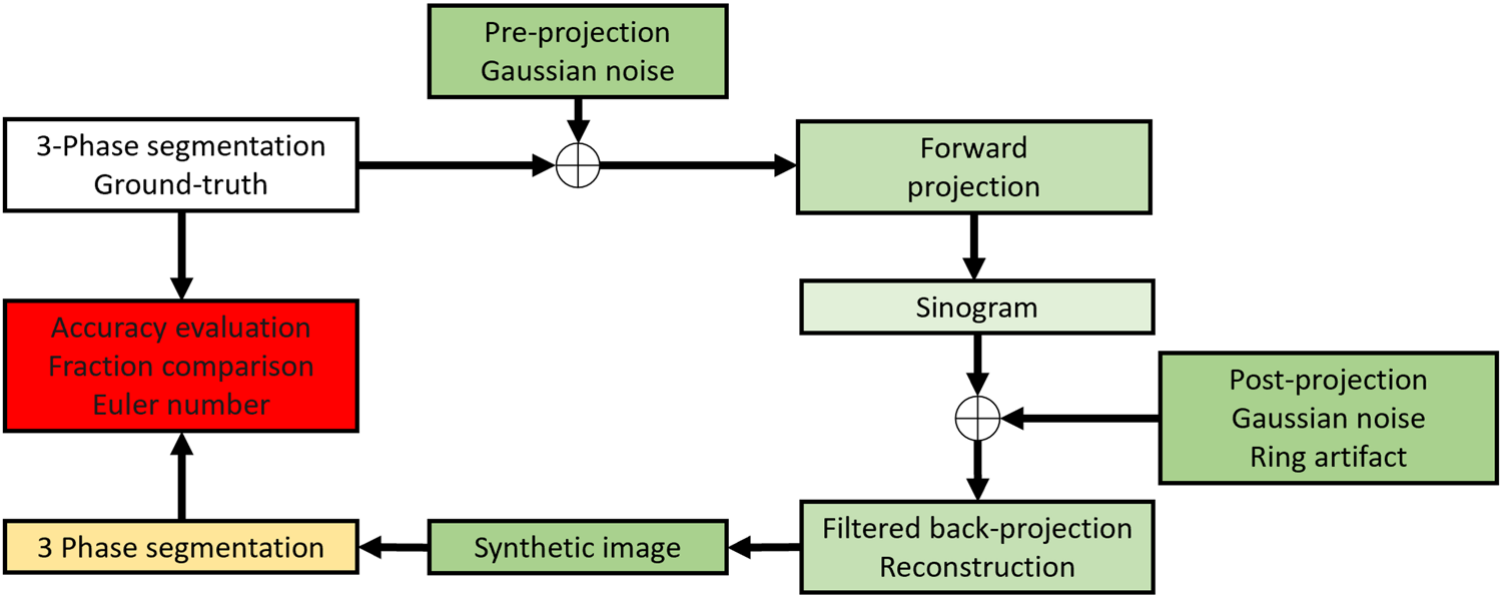
Synthetic image generation workflow using the Astra toolbox^33^. This workflow allows us to control the noise/artifacts level on the generated images.

**Figure 3:**
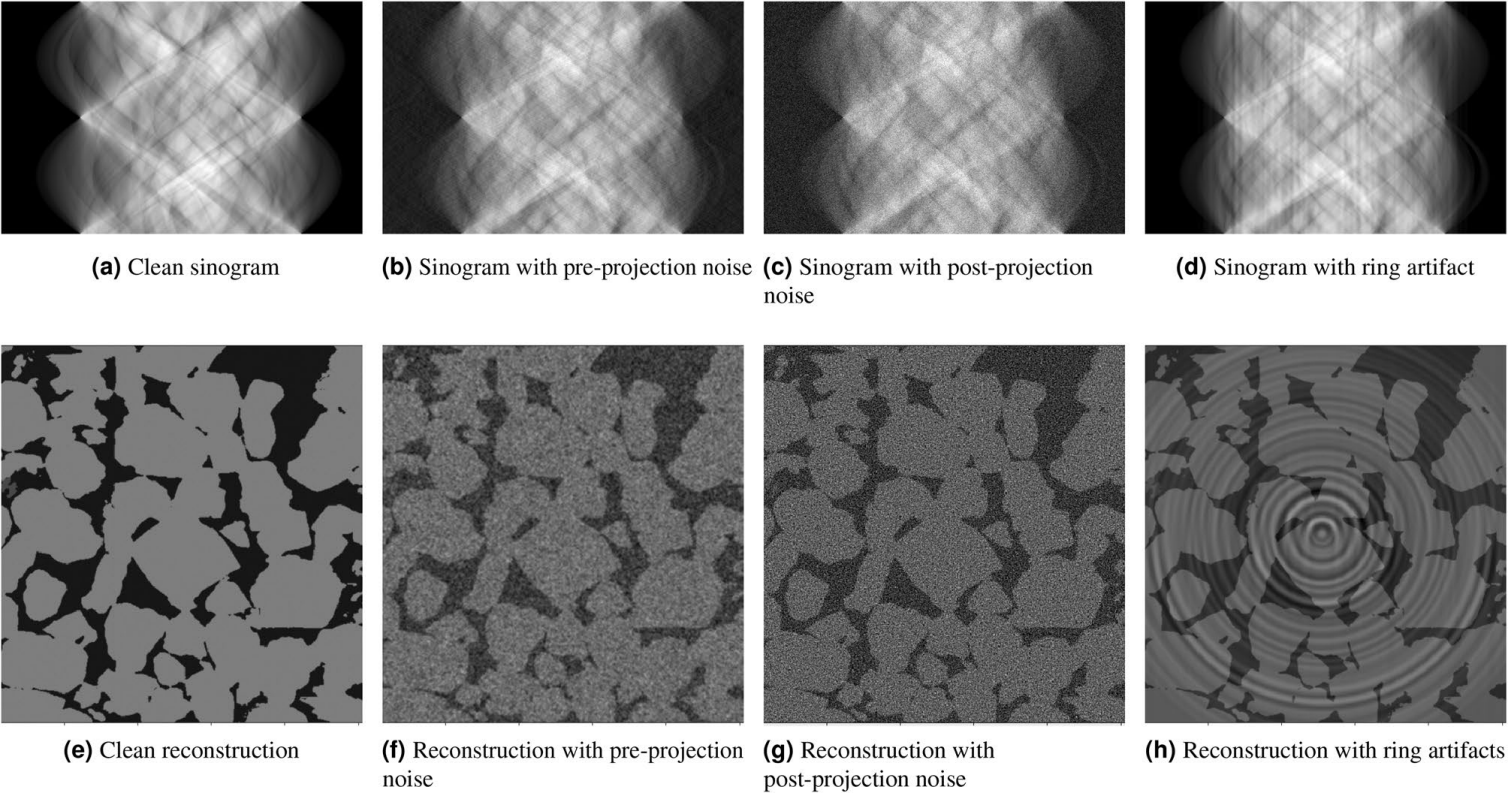
An illustration of different types of noise at the projection step (sinograms) and after the reconstruction step (micro-CT images). The ring artifacts appear as vertical stripes in the sinogram domain (**d**).

In order to evaluate the performance of our segmentation tool, we have used synthetic generated data from known ground truth images. We followed an existing workflow^32^ for generating synthetic gray-scale images using the Astra-toolbox^33^. Figure 2 shows the generation/validation workflow, where the ground truth segmented data get projected and reconstructed with filtered back projection in a parallel-beam geometry using a Ram-Lak filter. In addition, we can perform a proper noise sensitivity analysis since this workflow allows us to add different types of noise during the projection and reconstruction processes, reflecting the nature of noises/artifacts in real images. Figure 3 shows four examples of reconstructions from sinograms with different types of noise/artifacts.

## Results

### Comparison to traditional methods

**Figure 4:**
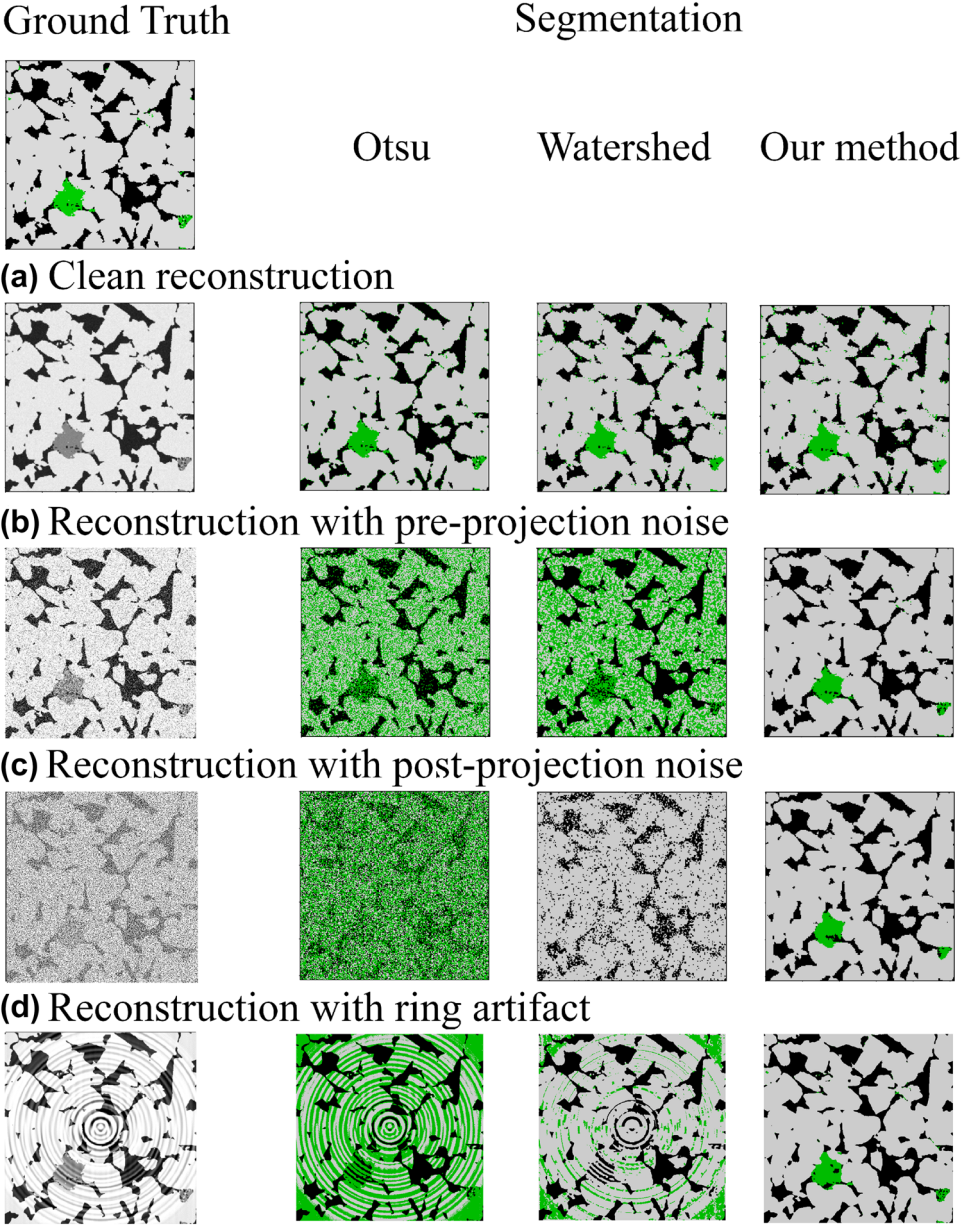
Comparison of segmentation results between Otsu, watershed and our method on four synthetic images corresponding to: (**a**) clean filtered back reconstruction, (**b**) reconstruction with noise pre-projection, (**c**) reconstruction with noise post-projection, and (**d**) reconstruction with ring artifacts.

We have generated several synthetic gray-scale images from real segmented ground truth images by adding different types of noise in the generation process, as described in the previous section. Figure 4 shows an example of four synthetic reconstruction cases: (a) clean reconstruction without noise, (b) reconstruction with Gaussian noise pre-projection, (c) reconstruction with Gaussian noise post-projection, (d) reconstruction with ring artifacts. For these four cases, we compare the results from our method to conventional segmentation methods: multi-Otsu thresholding and Marker-controlled Watershed. In this figure we show the middle slice of the 3D segmented images. The calculated accuracy and geometrical properties for all these cases are presented in Table 2. For each phase we have measured the total voxel-wise *accuracy*, *IOU* (Intersection Over Union). We have also calculated the *Euler number* (or Euler characteristic), a topological invariant that describes an object in a topological way regardless of the way it bent, and the *connected components count*, i.e the number of connected regions, using a 26-neighborhood connection algorithm^34^.

**Table 2:** Comparison of our model and traditional segmentation methods for several synthetic cases: (a) no noise, (b) pre-projection noise, (c) post-projection noise, and (d) ring artifacts.

Method	Accuracy (%)	IoU (%)			Fraction (%)			Euler number		Connected components count		
	Voxel-wise	Pore	Micro	Solid	Pore	Micro	Solid	Solid	Solid+Micro	Pore	Micro	Solid
Ground truth	100	100	100	100	21.17	0.98	77.85	− 1688	− 251	1172	1384	362
**Segmentation on clean reconstruction (Fig.**4a)												
Multi-Otsu	98.51	**99.76**	39.52	98.15	**21.13**	2.45	76.42	− 1348	− 803	1066	44,408	440
Watershed	98.46	96.09	39.14	98.96	20.45	2.28	77.27	− 4066	− 3618	192	54,787	44
Our method	**99.57**	98.19	**87.09**	**99.56**	21.26	**0.94**	**77.80**	**− 1383**	**− 332**	**1138**	**2533**	**420**
**Segmentation on reconstruction with pre-projection noise (Fig.**4b)												
Multi-Otsu	58.43	81.25	1.50	50.44	20.11	40.52	39.37	− 5,616,936	1,197,309	998090	204,586	25,750
Watershed	55.66	94.75	1.34	44.02	**21.47**	44.09	34.44	− 662,465	1487	4861	**2768**	2585
Our method	**98.75**	**95.26**	**66.95**	**98.65**	21.87	**0.90**	**77.23**	**− 1189**	**− 546**	**714**	6835	**114**
**Segmentation on reconstruction with post-projection noise (Fig. **4c)												
Multi-Otsu	35.29	26.09	0.99	31.00	30.27	42.07	27.66	− 10,789,612	5,208,879	63,995	36	204,920
Watershed	87.26	54.68	0.0	85.40	19.88	0.00	80.12	34,406	34,406	145,382	0	2030
Our method	**97.66**	**90.56**	**56.56**	**97.38**	**22.22**	**0.72**	**77.06**	**− 914**	**4421**	**2176**	**693**	**21**
**Segmentation on reconstruction with ring artifact (Fig. **4d)												
Multi-Otsu	57.010	92.10	1.43	46.04	**21.67**	42.45	35.88	− 28,630	2336	4168	16,944	48,242
Watershed	83.89	89.53	0.010	80.16	22.04	13.16	64.78	− 16,228	**− 2113**	692	13,616	2213
Our method	**98.23**	**93.31**	**49.35**	**98.22**	22.54	**0.92**	**76.54**	**− 1068**	2458	**1569**	**5038**	**231**

The closer the values to the ground truth the better. Bold values indicate the closest value to the ground truth in each category.

All methods are able to produce a correct segmentation on the noise-free case, Fig. 4a. However, it is interesting noticing that our method already produced closer Euler and connected counts to the ground truth. The micro-phase region is more disconnected in the case of multi-Otsu and Watershed segmentation. In the next case, Fig. 4b (i.e. Gaussian noise pre-projection), the results show a significant loss in accuracy for Otsu (58.43%) and watershed (55.66%) methods. These methods are very sensitive to Gaussian noise since multi-Otsu is based on global thresholding and watershed is based on neighbor gray-scale values. On the other hand, our method has shown a minor reduction in terms of voxel-wise accuracy (98.75%) and IOU, mostly due to the blurred border between the different phases. In the third case, Fig. 4 (i.e. Gaussian noise post-projection), the results show a significant variation in the fraction of each phase for conventional methods, even for open porosity which was relatively stable in the previous case. Despite the significant increment in the level of noise, our model showed 2% accuracy loss in terms of global accuracy, mostly due to the contrast reduction of interfaces, especially for micro-phase regions. This leads to a significant loss in IOU for the micro-phase, going down to 56%. However, as observed visually, this is a small error due to the small amount of micro-phase in the image (less than 1%). Finally in Fig. 4d, we show the last case (i.e. ring artefacts). The results are similar to the previous cases where multi-Otsu and watershed are really sensitive to the introduced noise performing very badly. As expected, the IOU of micro-phase for our method is lower than for the clean image (49.35%). As observer in this figure, the big local contrast of the gray values makes it harder to identify micro-phase regions. In general, these four cases are a good visual and qualitative description of how robust this type of methods is compared to conventional methods.

### Noise sensitivity analysis

**Figure 5:**
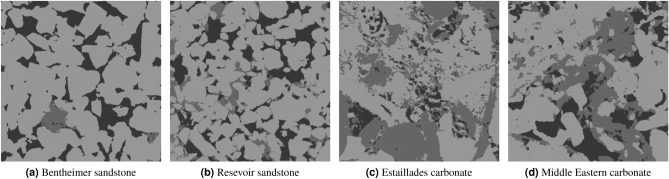
The four clean reconstructed synthetic images used for the noise sensitivity analysis.

**Figure 6:**
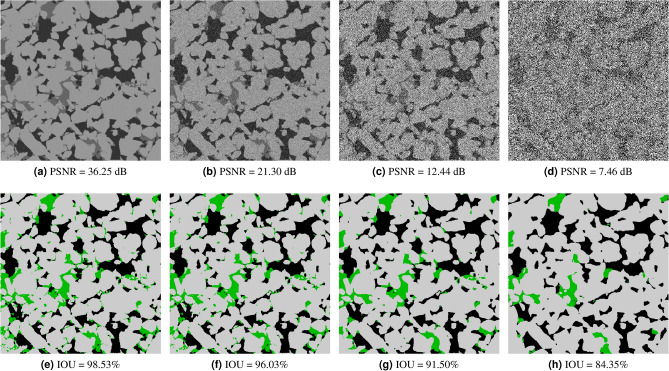
Segmentation results for the *reservoir sandstone* on synthetic images generated with Gaussian noise pre-projection.

**Figure 7:**
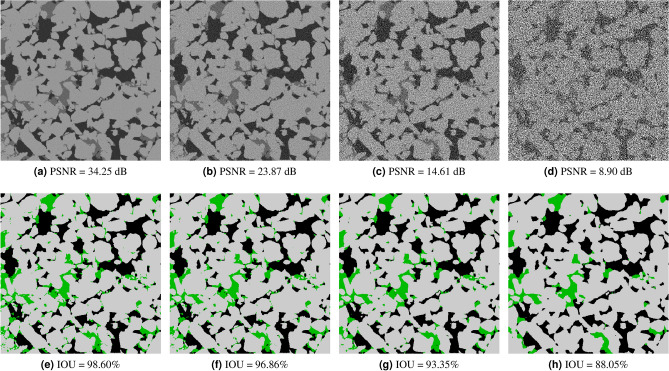
Segmentation results for the *Reservoir sandstone* on synthetic images generated with Gaussian noise post-projection.

**Figure 8:**
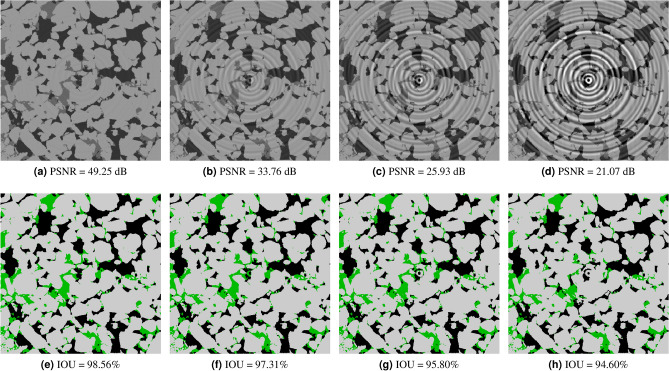
Segmentation results for the *reservoir sandstone* on synthetic images generated with ring artifacts.

In this section, we analyse the accuracy of our model to different types of synthetic noise and artifacts for four different rock samples: a bentheimer sandstone, a reservoir sandstone, an estaillades carbonate, and a middle eastern carbonate. In Fig. 5, we can see a synthetic reconstruction of each of these samples without noise. Additionally, we present pore, micro-phase and solid fractions in Table 3. For each rock image and each noise type we have generated around 50 synthetic new images with different noise levels. We have then processed all of them with our segmentation model and measured the accuracy respect to the ground truth. In the Figs. 6, 7 and 8 we show the Reservoir Sandstone gray-scale image and its corresponding segmentation at four different noise levels for Gaussian noise pre-projection, Gaussian noise post-projection, and ring artifact, correspondingly. As shown, in all the cases the model results do not break, even when the level of noise goes far beyond realistic noise levels, as shown in Fig. 7d. To the authors knowledge there is no segmentation model or algorithm in the literature reporting this level of robustness to noise.

**Table 3:** Fraction distribution for the different rock images used in the noise sensitivty analysis.

Rock type	Fraction (%)			Specific surface area		
	Pore	Micro	Solid	Pore	Micro	Solid
Bentheimer sandstone	21.69	1.01	77.30	0.284	0.770	0.078
Reservoir sandstone	18.21	9.71	72.08	0.433	0.927	0.165
Estaillades carbonate	5.40	42.22	52.38	0.836	0.552	0.276
Middle Eastern carbonate	12.99	23.07	63.94	0.438	0.599	0.166

**Figure 9:**
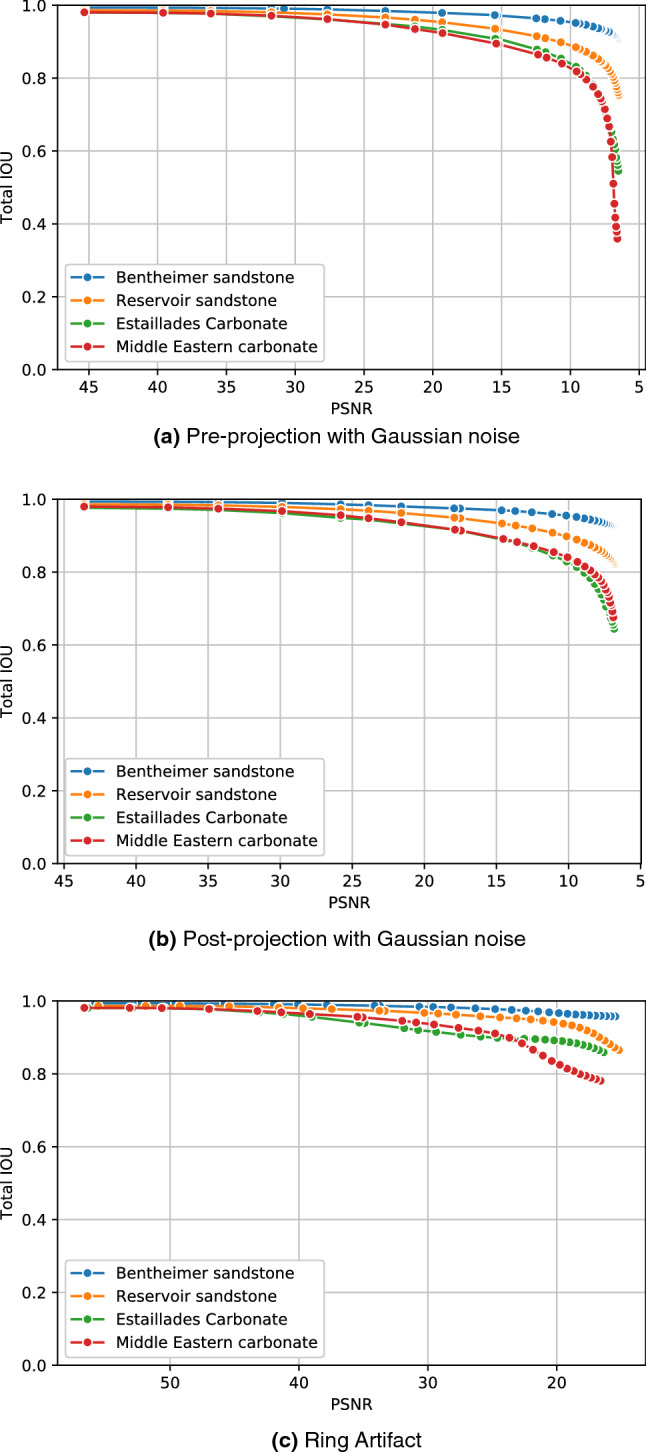
Segmentation sensitivity to different levels of Gaussian noise and ring artifact for four different rocks.

To quantify the level of noise introduced in each image we have used PSNR (Peak signal to noise ratio) measured in 8 bits. The PSNR between a reference image *f* and a test image *g* of size (*X*, *Y*, *Z*) is defined as:

1 $$\begin{aligned} {\text {PSNR}}(f, g)=10 \log _{10}\left( 255^{2} / {\text {MSE}}(f, g)\right), \end{aligned}$$

where

2 $$\begin{aligned} {\text {MSE}}(f, g)=\frac{1}{XYZ} \sum _{i=1}^{X} \sum _{j=1}^{Y} \sum _{k=1}^{Z}\left( f_{i j k}-g_{i j k}\right) ^{2}, \end{aligned}$$

so PSNR is an inverse logarithmic scale measured in decibels. The accuracy (IOU) vs. noise level (PSNR) curves for all these cases are shown in Fig. 9. As expected, the accuracy of the segmentation decreases monotonically for increasing levels of noise (lower PSNR). For the sandstone cases, with well-defined grains and pores, we observed that micro-phase is the most sensitive to noise and artifacts, while the accuracy of solid and pore are relatively stable even for extreme noise cases. So as expected, the larger the specific surface of a phase the more sensitive to noise this phase is. Accordingly, for the synthetic carbonate cases, without well-defined grains, the results show a slightly lower accuracy for pore than micro-phase regions. We observed that in these cases, the pores regions are more sensitive to noise than micro-phase regions. In general, for all types of noise we observe three different behaviours: *no-influence*, *correlated-noise* and *model-breakage*. For example, for Gaussian noise pre-projection, the inflection point between no-influence occurs at around PSNR = 35 dB and the model-breakage starts at around PSNR = 8 dB. In the first region it is hard to visually perceive any difference between segmented image and ground truth. In the second region, *correlated-noise*, we observe differences mainly located at the interfaces, starting from single voxels and small clusters. In the third region, *model-breakage*, one of the phases is affected significantly due to the added noise, most of the information defining the interfaces is missing. For this last region, small increases on the noise level produce significant accuracy loss. As observed in Fig. 7d,h, even for these extreme noise levels the solution maintains its coherence with the ground truth. When it comes to dealing with ring artifacts, Fig. 9c, it is important noticing that PSNR is less sensitive to these non-local artifacts. This means that for segmentations with similar IOUs, ring artifacts give a bigger visual effect than Gaussian noise. In these cases, as observed in Fig. 8, most of the error is concentrated at the center of the image where the information of local structure is completely missed.

In general terms, these results show that when trained appropriately this type of methods are extremely robust dealing with different types of noise and artifacts. In the next sections, we test these capabilities by evaluating the model for several types of real rock images and by comparing its behaviour to state-of-the-art human-assisted segmented images.

### Results on real images

#### Comparison to human-assisted segmentation

Due to the difficulty of defining an objective ground truth for real images, in this section we have directed our focus on comparing the performance of our model with respect to several human-assisted segmentations using traditional processing workflows. In addition, we investigate the variation of human-assisted segmentations and evaluate its effect on derived properties. We have used two images corresponding to Bentheimer and Berea sandstones. These images are challenging due to local variations in the gray-scale values. However, these images are representative of the general type and level of noises found in micro-CT imaging. We have provided the raw images (as produced by the scanners) to 5 different expert users. Each user chose the tools and workflows they considered more relevant to solve the assigned task. All the users have performed some filtering steps before segmenting the images. They have used conventional filters such as anisotropic diffusion^35^, beam hardening correction, median filter^36^, non-local means filter^37^ and ring artifacts removal; and segmentation methods such as multi-thresholding and marker-based watershed. They used implementations from open source and commercial packages such as: Avizo (TFS), Pergeos (TFS), ImageJ (open) and Mango (ANU).

**Figure 10:**
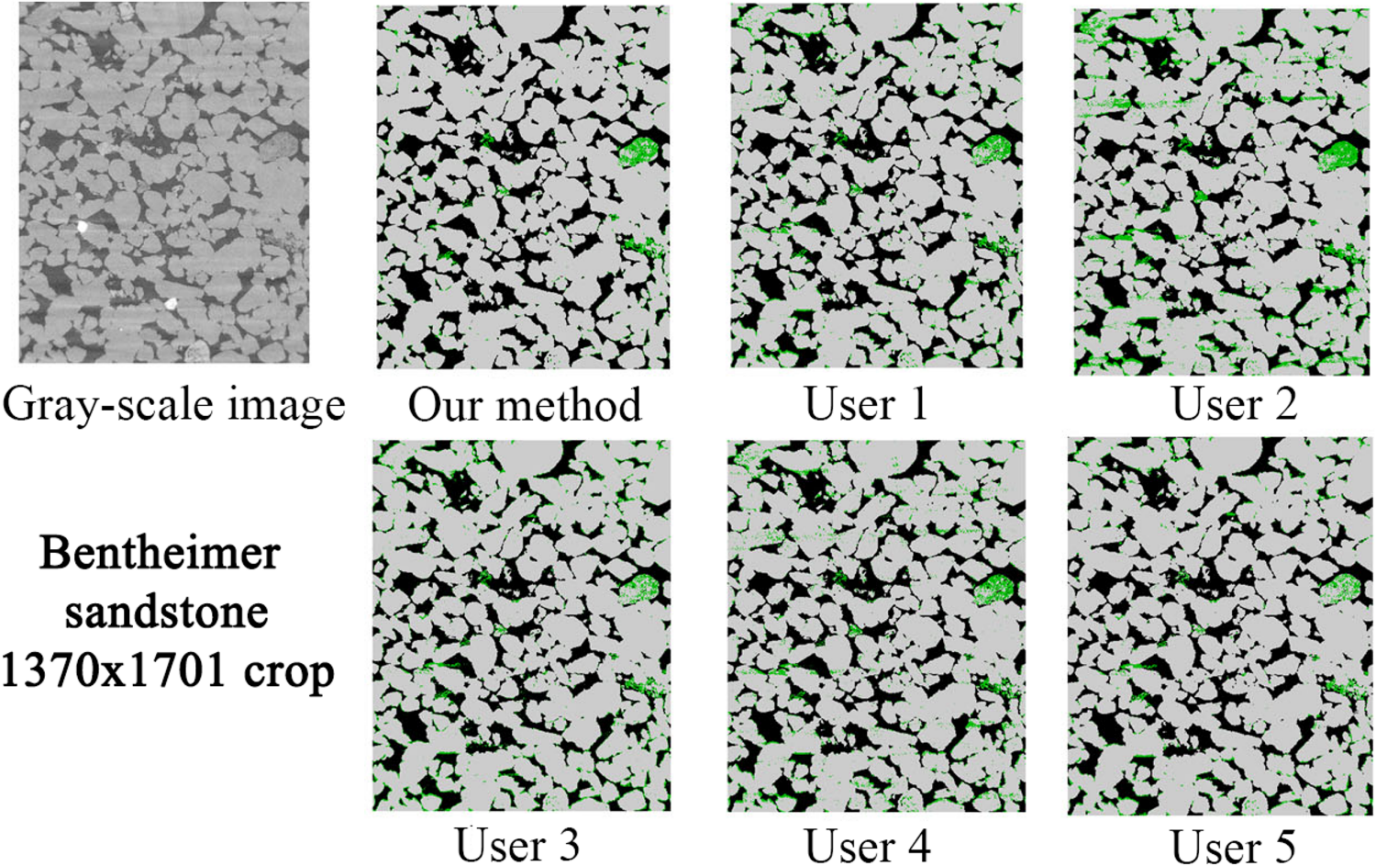
Comparison of segmentation results on a vertical full-size crop of the Bentheimer image. A significant level of noise in the form of stripes that span the width of the image, together with beam hardening can be seen in the bottom of the gray-image crop.

**Figure 11:**
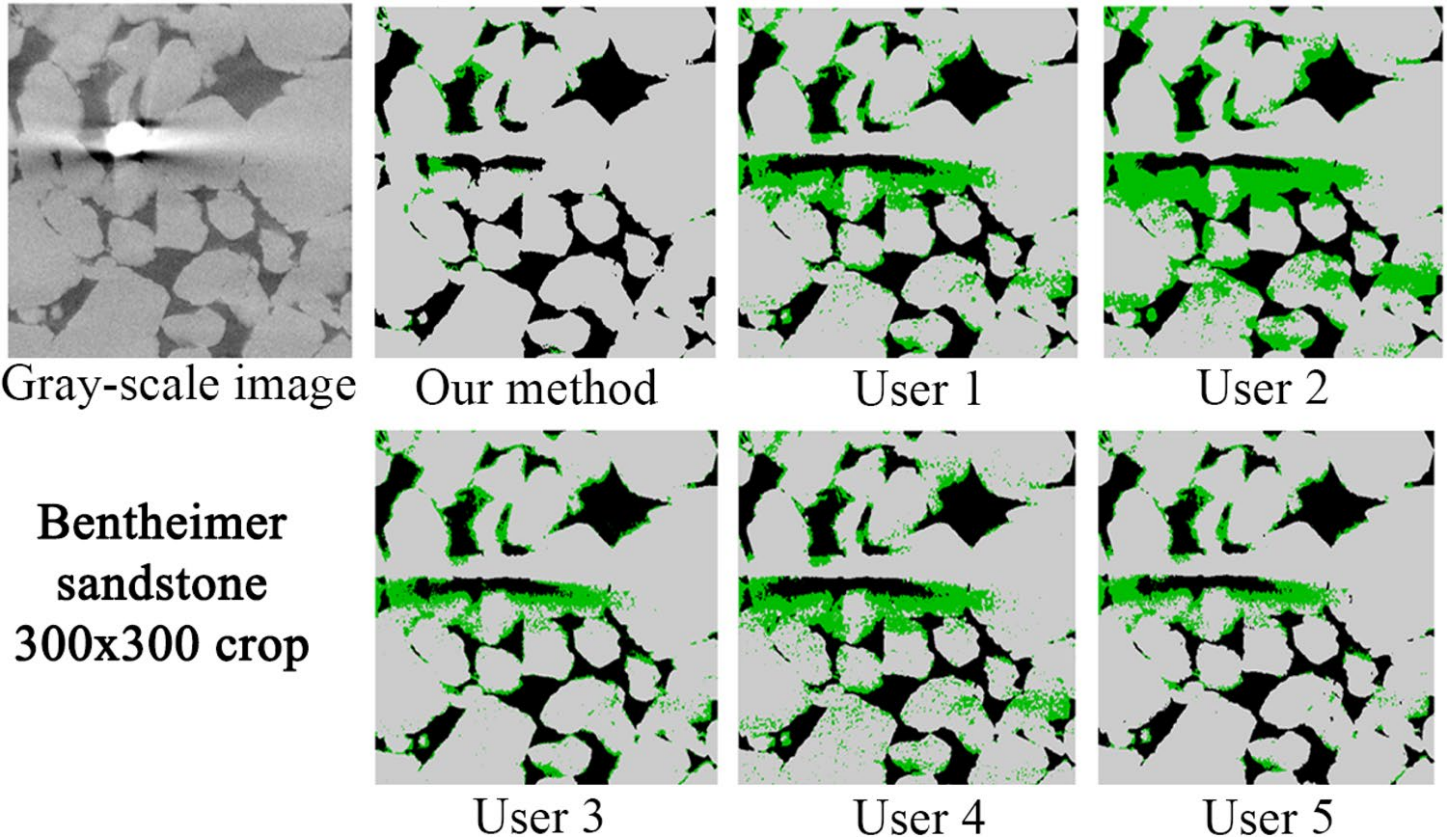
Comparison of segmentation results on a small crop of the Bentheimer image that was heavily affected by beam hardening.

Figure 10 shows the middle slice of the Bentheimer image and the corresponding segmentations from our model and the five users. In general terms, the two more severe problems in dealing with this image are the typical micro-CT stripes in Z direction (local gray-scale variations) and the high-density mineral artifacts. As observed, all the human-assisted segmentations seem to be very sensitive to these types of noise. This is reflected in a non-neglectful over-estimation of the micro-phase regions, which evidently due to the stochastic nature of the noise, it is not uniform for the different users. The segmentations corresponding to users 2 and 4 are clear examples of this problem. In addition, user 3 seems to have over-segmented the micro-phase in the interfaces between solid and pore. Figure 11 shows a detailed region of this image that has been heavily affected by high-density mineral artifact (local beam hardening). In this particular case, all the human-assisted segmentations reflect the second main problem, mentioned above. For all of the human-assisted segmentation cases we observe a significant over-estimation of the micro-phase around the high-density mineral regions due to a sharp shift in local brightness. In contrast, the segmentation of the model seems to handle these types of noise very well, even if not trained with this type of specific noise.

**Figure 12:**
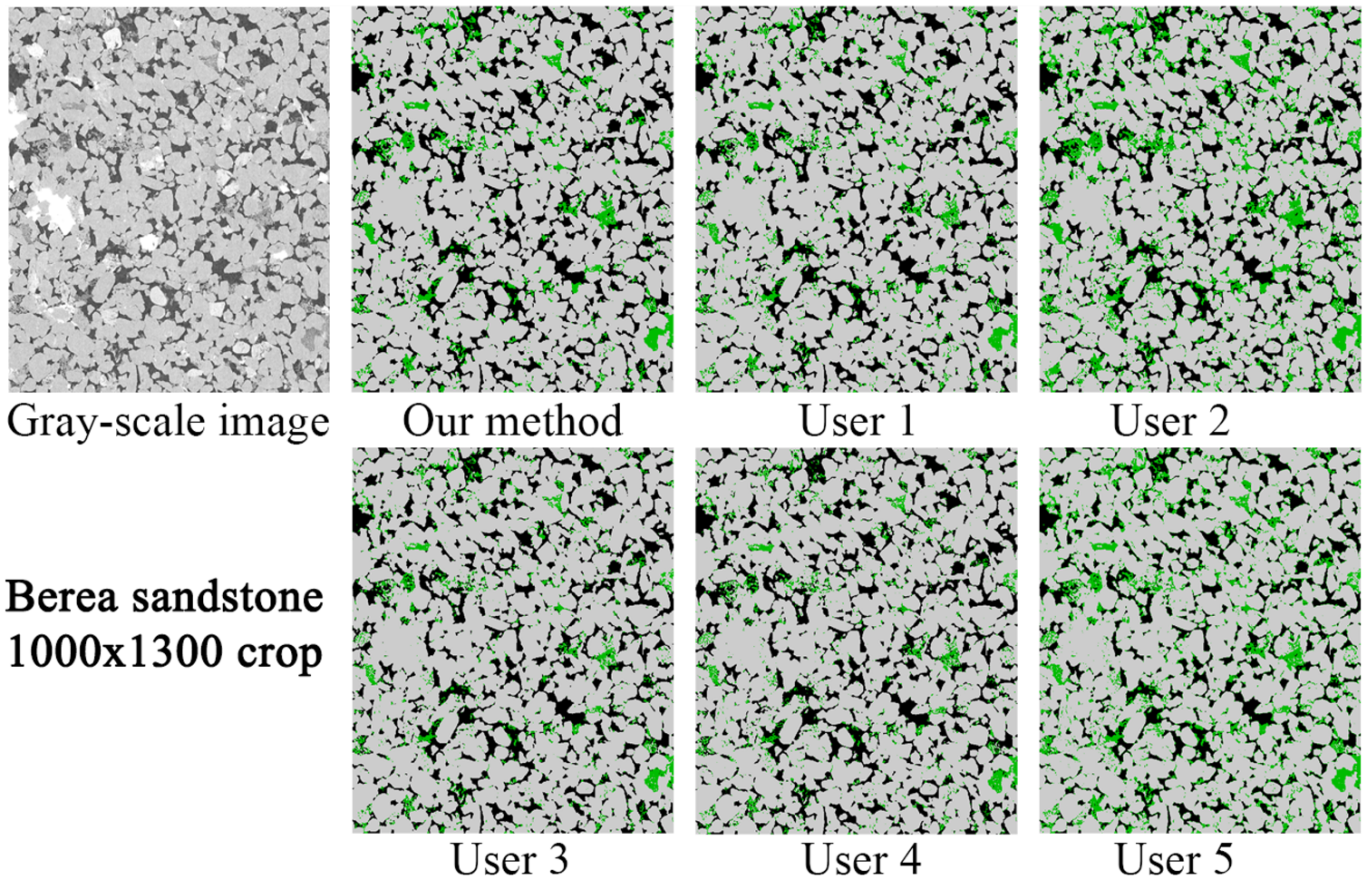
Comparison of segmentation results on a vertical top-half crop of the Berea image where we can see a significant variation in the amount of micro-phase between the different users.

**Figure 13:**
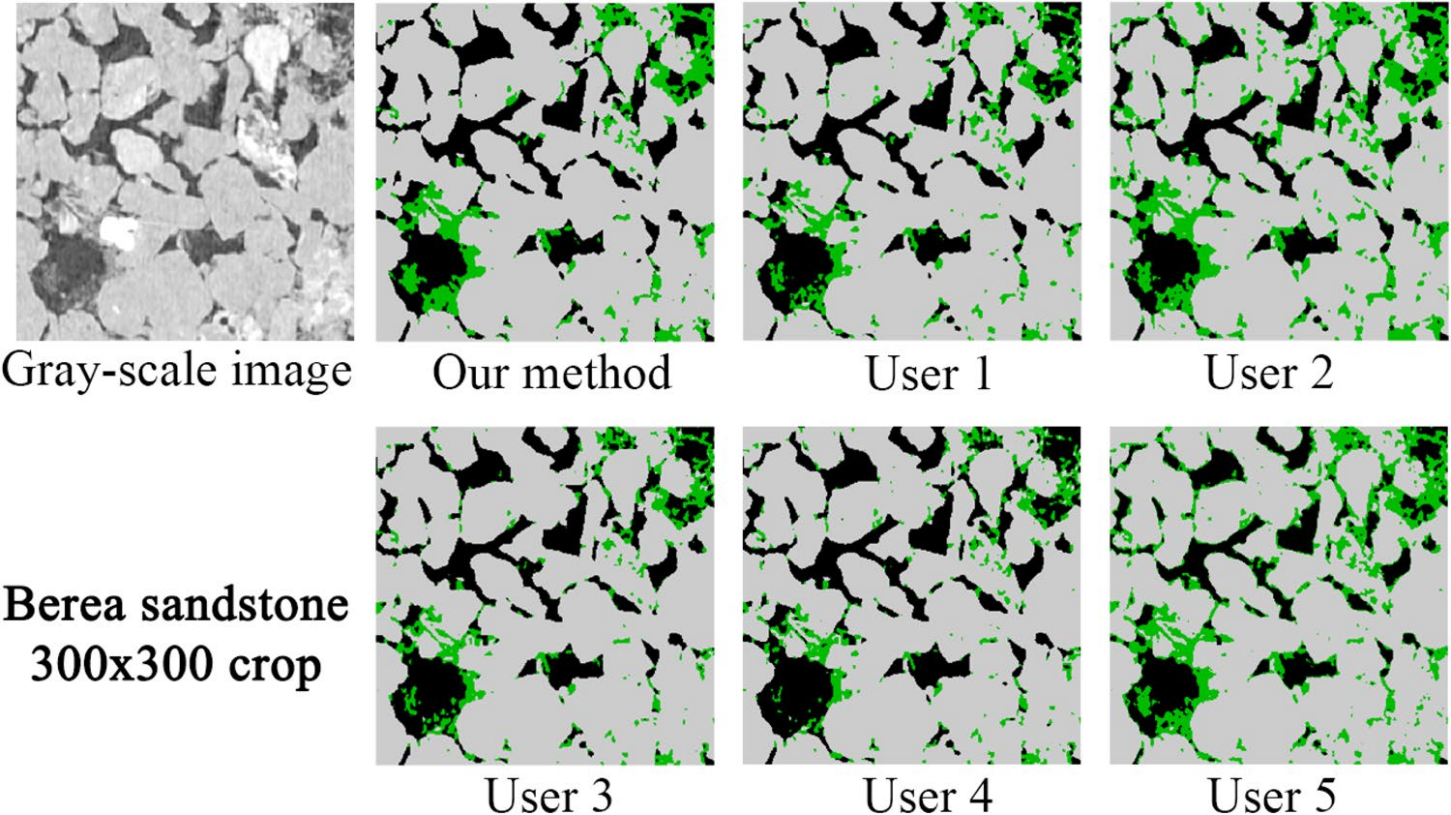
Comparison of segmentation results on a vertical top-half crop of the Berea image where we can see a significant different in the distribution of segmented micro-phase, especially inside the grain regions.

Figure 12 shows a slide of the gray-scale Berea image and the corresponding segmentation images. In terms of quality, this image has less noise and no significant artifact compared to the Bentheimer case. Figure 13 shows a more detailed crop where we can see a non-neglectful variation in the micro-phase volume for each segmentation. In Table 4, we summarized the measured and calculated properties on these segmented images for each of the users and our model. The calculated properties (i.e. permeability, formation factor and tortuosity) are calculated using a pore network model technique^2,38^. As we can see, the differences in the segmentations are reflected in all the properties. A significant variation of the results between the users is observed, most notably for the micro-porosity fraction and permeability, which is a common problem when working with real images as reported in the past^39^.

In the case of the Bentheimer sandstone, our method produced less micro-phase volume compared to the users, which is mainly due to its robustness to noise and artifacts as mentioned above. Our segmentation also has larger permeability compared to the users. This can be due to the larger open-pores (less micro-porosity) but also by the stripes of noise observed for most of the users, spanning the XY plane of the image. In this case, the users micro-phase volume covers a range of 3.51%. For Berea, the results from the users do not show a clear trend as previously (over-segmenting micro-phase). However, the variations of micro-phase volume are still quite significant ranging from 4.61 to 10.71%. Assuming a micro-phase volume as the users average, these variations translate to 75% variation for Bentheimer and a 86% variation for Berea (these numbers are worse assuming lower volumes). However, it is important to see that the model not only is more robust to noise but, additionally, it can help to reduce the human-usage variations since the model will perform equally independent of who is using it, since it does not require parameters tuning to do the segmentation work.

**Table 4:** Comparison of petrophysical properties between our method and human-assisted segmentations.

Type	u1	u2	u3	u4	u5	Users mean ± std	Our method
**Bentheimer sandstone (1370 × 1370 × 1701)**							
Open porosity (%)	21.66	19.48	19.77	21.89	20.93	20.74 ± 0.97	21.80
Micro porosity (%)	4.45	6.89	5.14	4.92	3.38	4.96 ± 1.14	2.54
Permability	2260	1574	1069	2294	1918	1823 ± 459	2319
Formation factor	13.14	17.33	13.06	12.24	15.84	14.32 ± 1.93	12.50
Tortuosity (min)	3.39	3.54	3.43	3.41	3.62	3.48 ± 0.09	3.30
**Berea sandstone (1000 × 1000 × 2399)**							
Open porosity (%)	17.73	17.29	18.25	18.89	15.00	17.43 ± 1.33	18.49
Micro porosity (%)	5.48	10.71	4.61	4.67	9.85	7.06 ± 2.66	5.25
Permability	712	643	746	841	394	667 ± 150	795
Formation factor	23.17	23.48	20.66	18.90	34.41	24.12 ± 5.41	18.82
Tortuosity (min)	3.58	3.55	3.47	3.44	3.57	3.52 ± 0.05	3.57

### Comparison to experimental results

**Figure 14:**
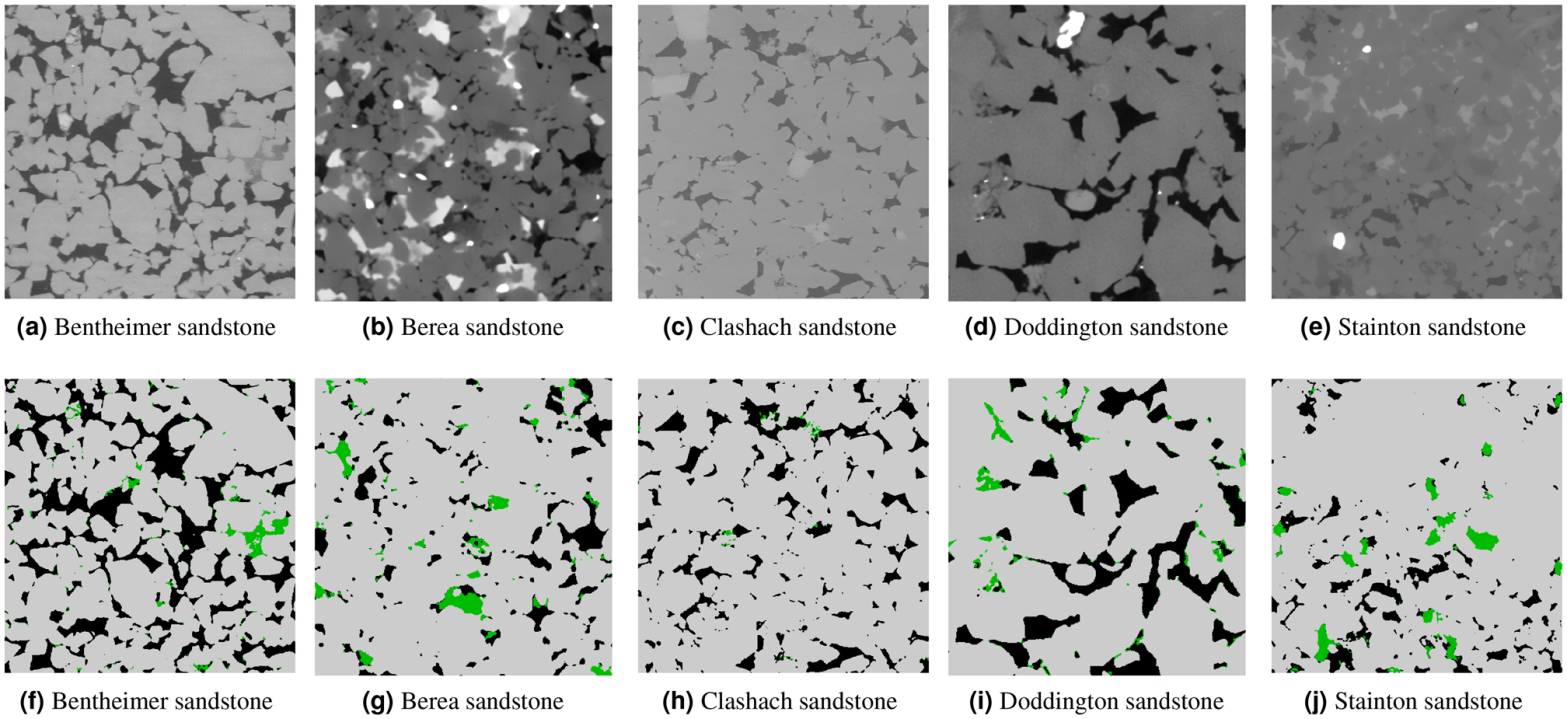
A full-size middle cut of 5 sandstone images and their corresponding three-phase segmentation by our model.

**Figure 15:**
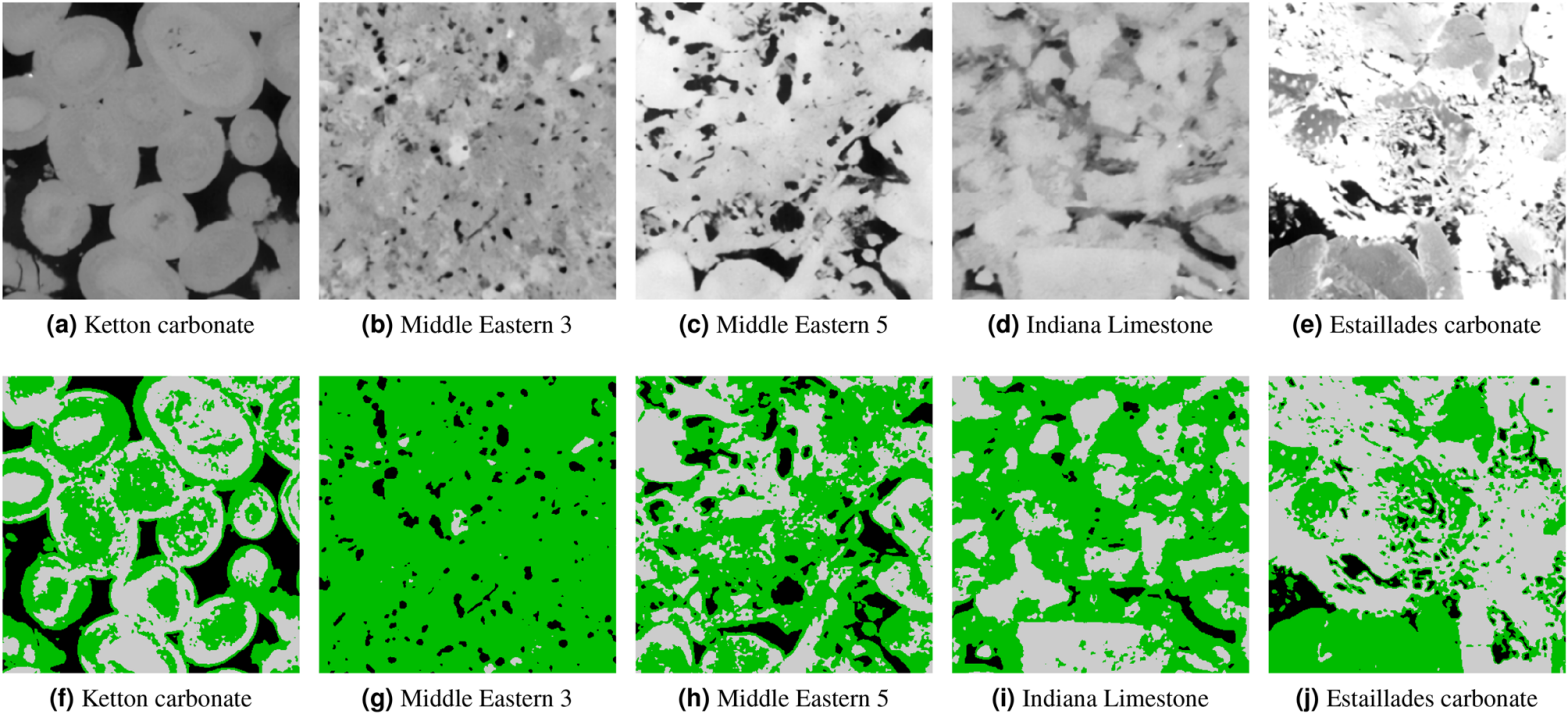
A full-size middle cut of 5 carbonate images and their corresponding three-phase segmentation by our model.

In this section, we compare the results of our model to porosities measured experimentally. We used the dataset presented in Shah et al.^40^, where the authors provided micro-CT images at different resolutions for 10 sandstone and carbonate samples and the corresponding experimental porosity, which was measured through the bulk volume by saturating the plugs with water. It is important to remark that these measurements were done on the whole cylindrical sample volume and the scanned region corresponds to a crop in the middle of the cylinder ($\sim$ 50%). However, as these rocks are relatively homogeneous it is expected that porosity values are in the same range. We used the images with the highest resolution of around 4 µm. These images are all 575 cubic voxels. Figures 14 and 15 show the middle slice of each image and the corresponding segmentation by our model. Table 5 shows the measured experimental porosity and the open and micro-phase fractions from our segmentation. For the sandstone cases, assuming a 50% porosity for micro-phase regions there is less than 1% difference compared to the experimental total porosity. For the carbonates, it is harder to assign a given porosity for the micro-phase since carbonates, in general, have a wider porosity distribution. For example, the Ketton segmentation, shown in Fig. 15f illustrates that most of the micro-phase regions have very close gray-scale value to the solid region and, thus, a lower porosity. Another clear example is the case of Middle Eastern 3 carbonate shown in Fig. 15g, where the micro-phase regions are covering the whole image (96%). In such cases, it would be necessary to use other types of imaging techniques, such as dry/wet based porosity map^28,29^, instead of a three-phase segmentation. For the rest of the carbonate cases, the segmentation performed by our model seems visually correct and the porosities are in the same range as the experiments.

**Table 5:** Comparison between experimental and our model segmentation porosity for several sandstone and carbonate micro-CT-images.

Type	Exp. porosity (%)	Calculated porosity (Our)	
		Open (%)	Micro (%)
**Sandstone**			
Bentheimer	19.0 ± 0.3	18.98	1.68
Berea	11.2 ± 0.4	10.52	1.83
Clashach	11.0 ± 0.2	11.65	0.36
Doddington	18.4 ± 0.5	17.37	2.05
Stainton	11.2 ± 0.4	9.49	3.24
**Carbonate**			
Ketton	18.9 ± 0.1	12.59	50.02
ME3	10.7 ± 0.4	6.69	86.59
ME5	24.8 ± 0.8	13.16	47.54
Indiana limestone	25.8 ± 0.5	3.20	52.93
Estaillades	22.6 ± 0.9	6.06	41.70

## Conclusion

We have developed a deep learning based three-phase segmentation model and trained it on multiple 3D micro-CT rock images with a wide range of domain-specific augmentation steps. We then studied our model’s performance on synthetic and real images in terms of accuracy and physical properties. Based on these results, it is clear that our segmentation model is capable of producing high-quality segmentations even when given noisy and low-quality input images. We have further validated our model’s performance with experimental results from other studies on multiple types of rocks. In addition, we compared the performance of our segmentation model to five different expert users. The segmentation results strongly indicated that the model is better at dealing with typical image noises compared to the expert users.

As a summary, the presented results demonstrate the potential capacity of a fully automated segmentation workflow, which would lead to a significant improvement on the image processing step compared to current processing workflows. It is worth mentioning that even if this study focuses on three-phase segmentation, it is relatively simple to extend the number of phases handled by the tool, given the availability of the necessary training data.
